# Characteristics of women who continue smoking during pregnancy: a cross-sectional study of pregnant women and new mothers in 15 European countries

**DOI:** 10.1186/1471-2393-14-213

**Published:** 2014-06-25

**Authors:** Janne Smedberg, Angela Lupattelli, Ann-Charlotte Mårdby, Hedvig Nordeng

**Affiliations:** 1 Department of Pharmacy, School of Pharmacy, University of Oslo, PO Box 1068, Blindern, 0316 Oslo, Norway; 2 Analysis Unit, Sahlgrenska University Hospital, Gothenburg, Sweden; 3 Division of Mental Health, Norwegian Institute of Public Health, Oslo, Norway

**Keywords:** Pregnancy, Smoking, Prevalence, Determinants, Europe

## Abstract

**Background:**

Some women continue smoking during pregnancy despite the extensive information available on the dangers smoking poses to their fetus. This study aimed to examine the prevalence and determinants of smoking before and during pregnancy and the extent of smoking during pregnancy from a European perspective in relation to maternal sociodemographic characteristics, health literacy, morbidity, and pregnancy-related factors.

**Methods:**

This multinational, web-based study evaluated pregnant women and new mothers in 15 European countries recruited from October 2011 to February 2012. Data were collected via an anonymous online questionnaire.

**Results:**

Of 8344 women included, 2944 (35.3%) reported smoking before pregnancy, and 771 (26.2%) continued smoking during pregnancy, 88 (11.4%) of whom smoked more than 10 cigarettes per day. There was a wide variation among the 15 European countries in smoking rates before and during pregnancy, ranging from 25.0% (Sweden) to 50.0% (Croatia) before and 4.2% (Iceland) to 18.9% (Croatia) during pregnancy. Women who lived in Eastern Europe, without a spouse/partner, with a low education level and unplanned pregnancy, who did not take folic acid, and consumed alcohol during pregnancy were the most likely to smoke before pregnancy. Women who lived in Eastern or Western Europe, without a spouse/partner, with a low education level and health literacy, being a housewife, having previous children and unplanned pregnancy, and who did not take folic acid were the most likely to continue smoking during pregnancy. Women who smoked more than 10 cigarettes per day during pregnancy were the most likely to be living in Eastern Europe and to have a low education level.

**Conclusion:**

Women with fewer resources living in Western or Eastern Europe are more likely not only to smoke before pregnancy but also to continue smoking during pregnancy. These high-risk women are characterized as living alone, having high school or less as highest education level, having low health literacy, being a housewife, having previous children, having unplanned pregnancy, and no use of folic acid. Our findings indicated that focus on smoking cessation is important in antenatal care in Europe as many women smoke before pregnancy, and still continue to do so in pregnancy.

## Background

Maternal smoking during pregnancy poses a significant threat to the unborn child. As there is no safe lower limit of cigarette use during pregnancy, the World Health Organization advises pregnant women to abstain from all cigarette use [1]. Women are more likely to stop smoking during pregnancy than at other times [2], yet some continue smoking despite the extensive information available on the dangers that smoking poses to their fetus. Smoking during pregnancy increases the risk of spontaneous pregnancy loss, preterm delivery, low birth weight (LBW) infant, small for gestational age infant, preterm premature rupture of membranes, placental abruption, placenta previa, and stillbirth [3-9]. A dose-related relationship between increased risk and heaviness of smoking has been shown for spontaneous pregnancy loss, LBW, placental abruption, and stillbirth.

It has been estimated that 10%-27% of the pregnant women in the European Union continue smoking during pregnancy [10]. Here it should be noted that the true prevalence of smoking during pregnancy may be difficult to discern, not only because some countries lack national statistics but also because of possible underreporting in studies relying on self-report [11]. Social norms discouraging smoking during pregnancy today may lead women to fail to disclose their true smoking status. Prevalence rates appear to have peaked and have begun to decline in most industrialized countries [12], whereas in other countries, such as in Eastern Europe and the Mediterranean area, smoking is becoming increasingly common among young women in general [13], and hence also possibly among pregnant women. Together with growing concern over smoking as an epidemic in the Third World in the future [14], smoking will continue to be one of the most important preventable risk factors for poor pregnancy outcomes.

The lack of sustained benefit from interventions during pregnancy [15-18] suggests that our understanding of the determinants of smoking during pregnancy remains inadequate. A large number of studies on possible sociodemographic predictors for smoking in pregnancy have been published internationally [19-24]. However, the lack of uniform study design, data collection, and study population make it difficult to assess whether these risk factors for smoking in pregnancy are similar in frequency and effects across countries. Furthermore, little is known about the impact of maternal morbidity and poor health literacy on smoking cessation when women who smoke become pregnant. In particular, there is a scarcity of comparable national data on smoking during pregnancy in Europe.

Hence, there is need for a study that simultaneously and uniformly collects data on smoking during pregnancy across nations. This can provide more insights into the burden of smoking during pregnancy in Europe and, not least, give clinicians and public health researchers useful information. Understanding what characterizes women who continue smoking during pregnancy would clearly lead to great potential health gains for both mother and child, and for society as a whole. The knowledge could be used to tailor preconception prevention strategies as well as interventions during pregnancy. Specifically, this knowledge is relevant for healthcare personnel (midwifes, doctors, and pharmacists) who offer smoking cessation support and sell nicotine replacement therapy to pregnant women who want to quit smoking.

The purpose of this study is to describe the prevalence and extent of smoking during pregnancy in 15 European countries in relation to maternal sociodemographic characteristics, health literacy, morbidity, and pregnancy-related factors. We used data from a multinational, cross-sectional, web-based study on pregnant women and new mothers.

## Methods

### Study design, population, and data collection

This is a sub-study of a multinational, cross-sectional, web-based study conducted in countries in Western, Northern, and Eastern Europe, South America, North America, and Australia, investigating medication use in pregnancy with focus on attitudes, perception of risk, and mental well-being [25]. For this study, we included only European countries (Austria, Croatia, Finland, France, Iceland, Italy, the Netherlands, Norway, Poland, Russia, Serbia, Slovenia, Sweden, Switzerland, and the United Kingdom). Both pregnant women at any gestational week and new mothers with a child younger than one year old were eligible to participate. Data were collected using an anonymous, self-completed, online questionnaire (http://www.questback.com) that was accessible for a period of 2 months in each participating country between October 2011 and February 2012.

The questionnaire was open to the public via utilization of banners (study invitation) on national websites and/or social networks commonly visited and consulted by pregnant women and/or new mothers. Information about recruitment tools utilized and Internet penetration rates in each participating country are described in detail elsewhere [25]. Here, the study’s external validity was assessed by comparing sociodemographic and lifestyle characteristics of the sample on an individual country level with those of the general birthing population in the country [25]. Overall, the birthing population in each participating country was reflected adequately by the sample with respect to age and smoking habits.

The questionnaire was first developed in Norwegian and English and then translated into the other relevant languages. A pilot study in four countries (n = 47) elicited no major changes. Collected data were searched for the presence of potential duplicates (based on reported country of residency, sociodemographics, date and time of questionnaire completion) but none were identified.

### Dependent variables

Smoking was the outcome variable, measured by the questions: ‘Did you smoke cigarettes before becoming pregnant?’ and ‘Do you/did you smoke during pregnancy?’ Women, who smoked before but not during pregnancy were classified as having quit smoking in pregnancy. Women who smoked before and during pregnancy were classified as continuing smoking in pregnancy.

Extent of smoking was measured by the question ‘How many cigarettes (on average) do you/did you smoke per day during pregnancy?’ The response alternatives were: ‘< 1,’ ‘1-5,’ ‘6-10,’ or ‘≥ 11’.

### Independent variables

Maternal sociodemographic characteristics included country of residency categorized according to the three regions Western Europe (Austria, France, Italy, Switzerland, the Netherlands, the United Kingdom), Northern Europe (Finland, Iceland, Norway, Sweden), and Eastern Europe (Croatia, Poland, Russia, Serbia, Slovenia), age, marital status, education level, working status, first language, and previous children. Pregnancy-related factors included planned pregnancy, folic acid use before and/or during pregnancy, and alcohol use during pregnancy. The variables were categorized as in Table 1.

**Table 1:** Maternal characteristics of non-smokers and smokers before pregnancy and of continuers and quitters during pregnancy

**Maternal characteristics**	***Total study sample (n = 8344)***			***Women smoking (n = 2944)***		
	**Women not smoking**	**Women smoking before pregnancy**	**p-value**	**Women who continued smoking when pregnant**	**Women who quit smoking when pregnant**	**p-value**
	**(n = 5400) (64.7%)**	**(n = 2944) (35.3%)**		**(n = 771) (26.2%)**	**(n = 2173) (73.8%)**	
	***n (%)***	***n (%)***		***n (%)***	***n (%)***	
**Region of residency**						
Western Europe^*^	2240 (41.5)	954 (32.4)	<0.001	277 (35.9)	677 (31.2)	0.014
Northern Europe^†^	1922 (35.6)	893 (30.3)		206 (26.7)	687 (31.6)	
Eastern Europe^§^	1238 (22.9)	1097 (37.3)		288 (37.4)	809 (37.2)	
**Maternal age**						
≤ 20	95 (1.8)	151 (5.1)	<0.001	51 (6.6)	100 (4.6)	0.051
21-30	2802 (51.9)	1777 (60.4)		459 (59.5)	1318 (60.7)	
31-40	2375 (44.0)	976 (33.2)		246 (31.9)	730 (33.6)	
> 40	128 (2.4)	40 (1.4)		15 (1.9)	25 (1.2)	
**Marital status**						
Married/cohabiting	5232 (96.9)	2694 (91.5)	<0.001	668 (86.6)	2026 (93.2)	<0.001
Other	168 (3.1)	250 (8.5)		103 (13.4)	147 (6.8)	
**Highest education level**						
Less than high school	147 (2.7)	220 (7.5)	<0.001	95 (12.3)	125 (5.8)	<0.001
High school	1360 (25.2)	1014 (34.4)		348 (45.1)	666 (30.6)	
More than high school	3260 (60.4)	1366 (46.4)		230 (29.8)	1136 (52.3)	
Other	633 (11.7)	344 (11.7)		98 (12.7)	246 (11.3)	
Health literacy^**^						
Low	264 (5.0)	215 (7.4)	<0.001	74 (9.8)	141 (6.6)	0.015
Medium	2113 (39.7)	1372 (47.4)		349 (46.3)	1023 (47.8)	
High	2943 (55.3)	1305 (45.1)		331 (43.9)	974 (45.6)	
**Working status**						
Employed (not as HCP)	3273 (60.7)	1755 (59.7)	<0.001	418 (54.4)	1337 (61.6)	<0.001
Healthcare personnel (HCP)	772 (14.3)	349 (11.9)		73 (9.5)	276 (12.7)	
Housewife	449 (8.3)	208 (7.1)		82 (10.7)	126 (5.8)	
Other	898 (16.7)	629 (21.4)		196 (25.5)	433 (19.9)	
**First language**						
Same as in country of residency	5071 (94.1)	2795 (95.2)	0.047	733 (95.3)	2062 (95.1)	0.894
Other	318 (5.9)	142 (4.8)		36 (4.7)	106 (4.9)	
**Previous children**						
No	2633 (48.8)	1617 (54.9)	<0.001	385 (49.9)	1232 (56.7)	0.001
Yes	2767 (51.2)	1327 (45.1)		386 (50.1)	941 (43.3)	
**Morbidity not incl. RD**^††^						
Yes	541 (18.4)	941 (17.4)	0.291	157 (20.4)	384 (17.7)	0.109
No	2403 (81.6)	4459 (82.6)		614 (79.6)	1789 (82.3)	
**Respiratory disease**^§§^						
Yes	306 (10.4)	603 (11.2)	0.296	71 (9.2)	235 (10.8)	0.235
No	2638 (89.6)	4797 (88.8)		700 (90.8)	1938 (89.2)	
**Planned pregnancy**						
Yes/not completely unexpected	5002 (93.0)	2606 (88.7)	<0.001	647 (84.0)	1959 (90.3)	<0.001
No, it was not planned	377 (7.0)	333 (11.3)		123 (16.0)	210 (9.7)	
Folic acid use^*†^						
Yes	5022 (93.5)	2600 (89.2)	<0.001	642 (84.9)	1958 (90.7)	<0.001
No	347 (6.5)	314 (10.8)		114 (15.1)	200 (9.3)	
**Alcohol consumption during pregnancy**						
Yes	790 (14.8)	527 (18.0)	<0.001	154 (20.1)	373 (17.3)	0.090
No	4556 (85.2)	2398 (82.0)		612 (79.9)	1786 (82.7)	

HCP = health care provider; RD = respiratory disease.

Numbers may not add up to total due to missing values. For *Folic acid use* and *Alcohol consumption during pregnancy*, the response ‘cannot remember’ was treated as a missing value. Missing values are less than 5% of the total.

^*^Includes Austria, France, Italy, Switzerland, the Netherlands, United Kingdom.

^†^Includes Finland, Iceland, Norway, Sweden.

^§^Includes Croatia, Poland, Russia, Serbia, Slovenia.

^**^Measured by a self-assessment scale comprising three questions corresponding to the set of brief screening questions (SBSQ) developed to detect inadequate or marginal health literacy in clinical settings [26,27].

^††^Includes the presence of a chronic disease (hypothyroidism, rheumatic diseases (incl. rheumatoid arthritis, psoriatic arthritis), diabetes (type I or II), epilepsy, depression, anxiety, and cardiovascular diseases (incl. hypertension, high cholesterol, and heart diseases) or others (open-ended option)).

^§§^Includes the presence of asthma, allergy, rhinitis, tonsillitis, sinusitis, or other respiratory tract conditions.

^*†^Indicates folic acid use before and/or during pregnancy.

Health literacy was measured by a self-assessment scale comprising three questions corresponding to the set of brief screening questions (SBSQ) developed to detect inadequate or marginal health literacy in clinical settings [26]: (1) ‘How often do you have someone help you read hospital materials?,’ (2) ‘How confident are you filling out medical forms by yourself?,’ and (3) ‘How often do you have problems learning about your medical condition because of difficulty understanding written information?’ We assigned zero (highest problems with reading or learning/not at all confident in filling out medical forms) to 4 points (no problems with reading or learning/extremely confident in filling out medical forms) to the scaled responses for the three questions. We then summed the scores to obtain a 0- or 12-point scale, with higher scores indicating higher health literacy level. The SBSQ sum score was trichotomized into low (score 0–5), medium (score 6–9), and high health literacy (score 10–12). The three brief screening questions have been found effective in detecting inadequate health literacy [26].

The morbidity variable included the presence of a chronic disease except for respiratory diseases (i.e., hypothyroidism, rheumatic diseases (including rheumatoid arthritis, psoriatic arthritis), diabetes (type I or II), epilepsy, depression, anxiety, and cardiovascular diseases (including hypertension, high cholesterol, and heart diseases). An additional open-ended option was included to capture diseases other than those specifically mentioned. The respiratory diseases variable included the presence of asthma, allergy, rhinitis, tonsillitis, sinusitis, or other respiratory tract conditions.

### Ethical approval

The study was approved by the Regional Ethics Committee South-East in Norway. Before obtaining access to the online questionnaire, each participant was asked to read the study description and then answer the question “Are you willing to participate in the study?” The participants gave informed consent by ticking the answer “yes” to this question. All data were handled and stored anonymously.

### Statistical analyses

Descriptive statistics were utilized as appropriate. The Pearson Chi square test was used to compare the maternal characteristics in smokers and non-smokers and in relation to the extent of smoking. The reporting of smoking was initially evaluated separately for pregnant women and new mothers. However, as there were no significant differences in reporting of smoking during pregnancy between pregnant women and new mothers or between pregnant women in their first (25.2%), second (26.6%), or third trimester (26.7%), the entire study population was used in the analyses of smoking cessation. Bivariate and multivariate logistic regression was used to explore determinants of smoking during pregnancy. A p value of < 0.05 was considered statistically significant. Data are presented as crude and adjusted odds ratios (OR) with 95% confidence intervals (CI). The multivariate model was built after fitting the bivariate logistic regression model for all explanatory variables. Purposeful selection of candidate variables was done based on a bivariate p value < 0.15. We then fit a reduced model by removing variables having no role (p value > 0.05), except if they yielded a change larger than 15% in the beta coefficients of the retained variables. The main effect model was checked for presence of interactions. The Hosmer and Lemeshow test was used to assess goodness of fit of the final multivariate model [28]. Sub-analyses on maternal status (pregnant or new mothers) were performed to check for recall bias, and sub-analyses on pregnancy status (trimester) were performed to check for inflation/reduction of smoking prevalence across the pregnancy. All statistical analyses were carried out using the Statistical Package for the Social Sciences (SPSS) version 20.0 (IBM® SPSS® Statistics).

## Results

### Population characteristics

**Figure 1:**
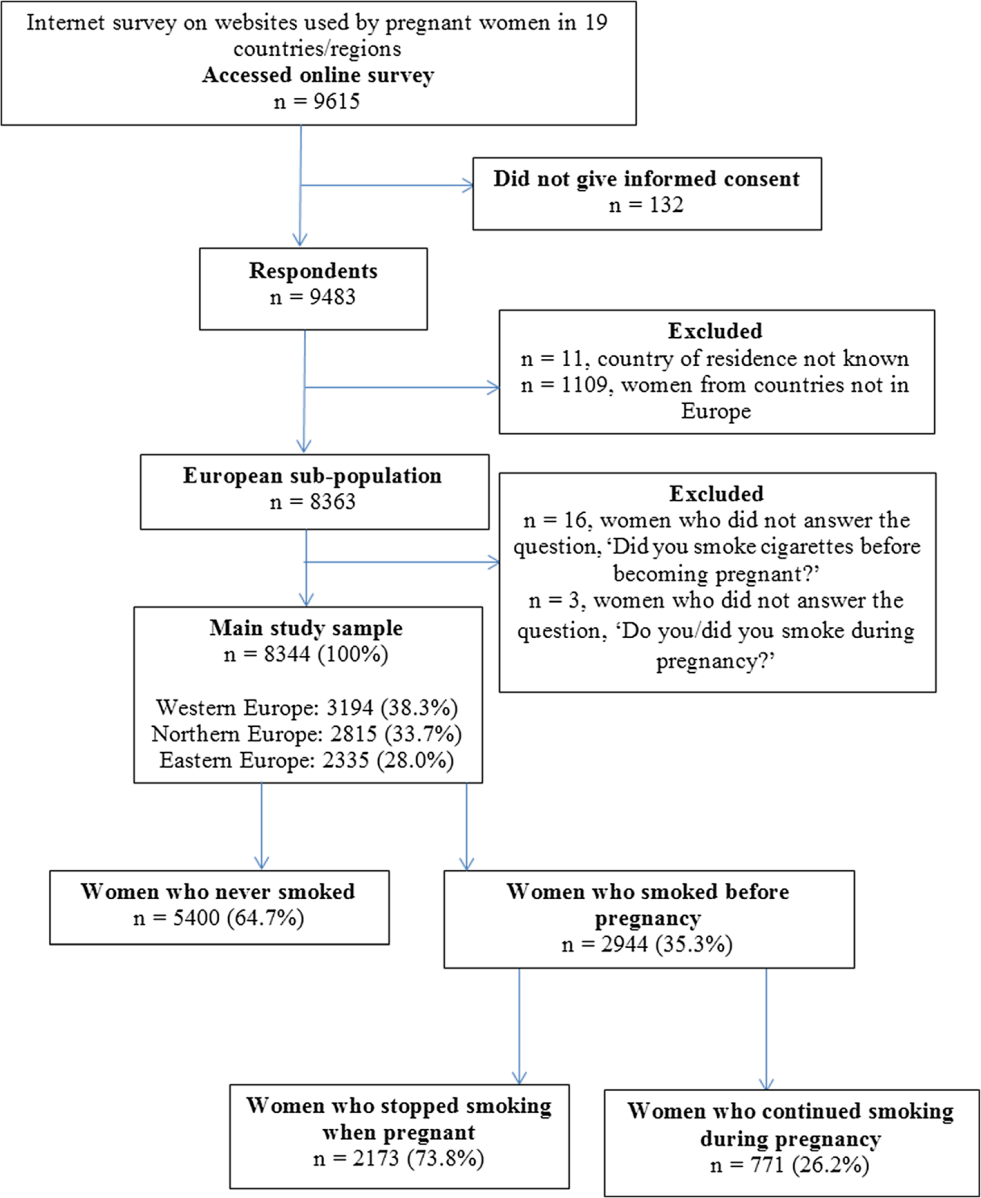
Participant flow-chart to achieve final analysis.

A total of 9615 women accessed the online questionnaire, whereof 9483 (98.6%) completed it. Women with no eligible country of residency and with residency outside Europe were excluded, leaving 8363 participants. Of these, 8344 women answered the smoking questions. Figure 1 shows the data selection carried out to achieve the final study sample.

**Figure 2:**
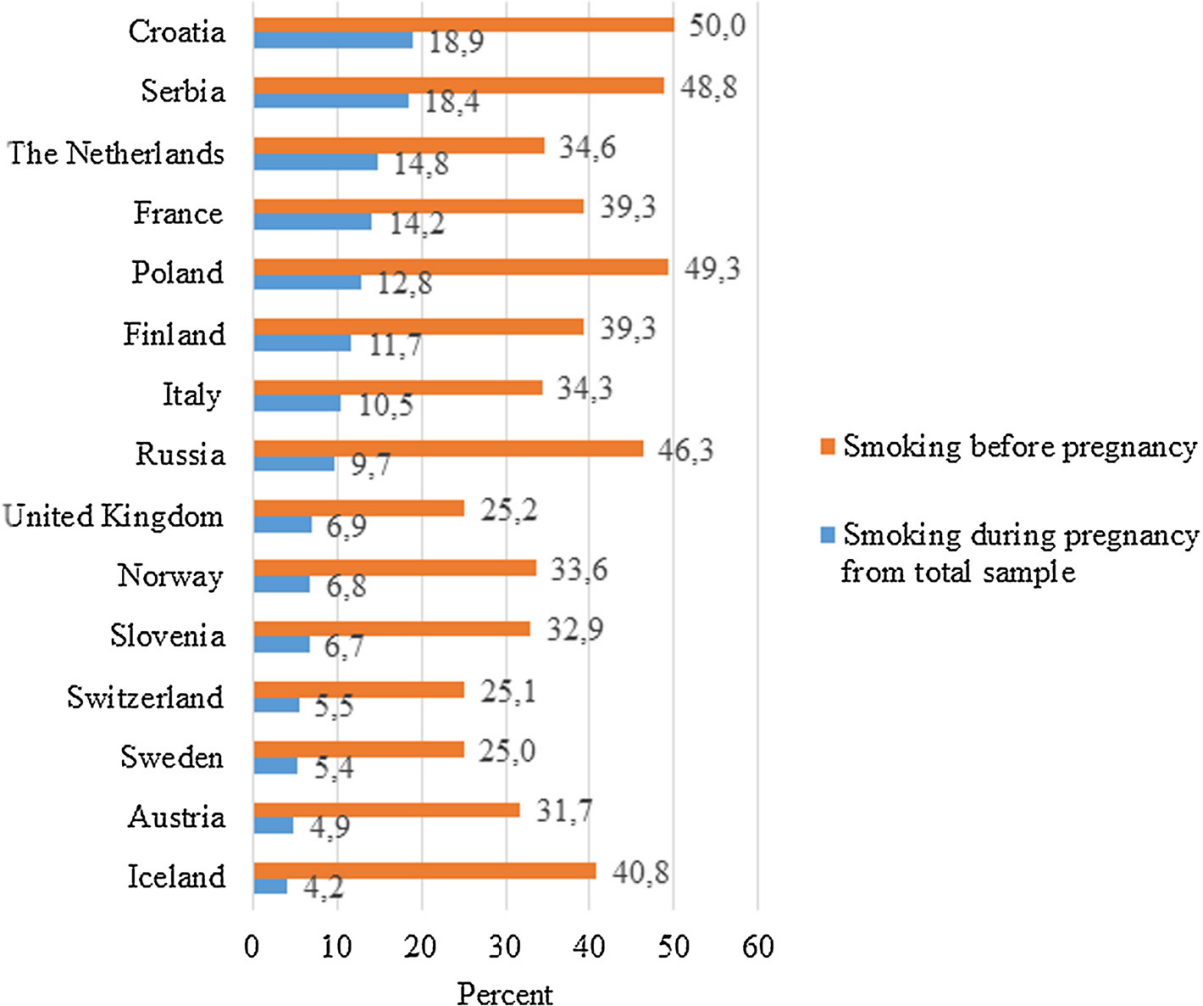
Prevalence of women smoking before pregnancy and of women who continue smoking during pregnancy in the countries of residency based on the total study sample (n = 8344).

Of the 8344 women who answered the smoking questions, 2944 (35.3%) reported smoking before pregnancy. Of these, 771 (26.2%) continued smoking during pregnancy. Mean gestational age was 22.3 weeks among non-smokers and 23.1 weeks among smokers before pregnancy (Mann–Whitney U test, p = 0.02) and 23.3 weeks among continuers and 23.0 weeks among quitters during pregnancy, (Mann–Whitney U test, p = 0.60). Figure 2 shows prevalence of smoking before and during pregnancy according to country, and Table 1 shows maternal characteristics of smokers and non-smokers before pregnancy and continuers and quitters during pregnancy. The prevalence of smoking before pregnancy ranged from 25.0% in Sweden to 50.0% in Croatia, and smoking during pregnancy ranged from 4.2% in Iceland to 18.9% in Croatia (Figure 2). Smokers before pregnancy and non-smokers significantly differed in region of residency, age, marital status, education level, health literacy, working status, first language, parity, pregnancy planning, folic acid use before and/or during pregnancy, and alcohol use during pregnancy (Table 1). Continuers and quitters during pregnancy significantly differed in region of residency, marital status, education level, health literacy, working status, parity, pregnancy planning, and folic acid use before and/or during pregnancy (Table 1).

Table 2 shows maternal characteristics according to the extent of smoking during pregnancy. Among the 771 women who continued smoking during pregnancy, 99 (12.8%) smoked less than one cigarette per day, 380 (49.3%) smoked 1–5 cigarettes per day, 203 (26.3%) smoked 6–10 cigarettes per day, and 88 (11.4%) smoked more than 10 cigarettes per day. The extent of smoking only differs significantly in relation to alcohol consumption during pregnancy.

**Table 2:** Maternal characteristics according to extent of smoking during pregnancy

**Maternal characteristics**	***Extent of smoking during pregnancy measured per day (n = 770)***^***¥***^				
	**< 1 cigarette (n = 99)**	**1-5 cigarettes (n = 380)**	**6-10 cigarettes (n = 203)**	**≥ 11 cigarettes (n = 88)**	**p-value**
	***n (%)***	***n (%)***	***n (%)***	***n (%)***	
**Region of residency**					
Western Europe^*^	37 (37.4)	135 (35.5)	78 (38.4)	27 (30.7)	0.068
Northern Europe^†^	36 (36.4)	98 (25.8)	53 (26.1)	19 (21.6)	
Eastern Europe^§^	26 (26.3)	147 (38.7)	72 (35.5)	42 (47.7)	
**Maternal age**					
≤ 20	8 (8.1)	25 (6.6)	17 (8.4)	1 (1.1)	0.293
21-30	61 (61.6)	227 (59.7)	117 (57.6)	53 (60.2)	
31-40	29 (29.3)	118 (31.1)	68 (33.5)	31 (35.2)	
> 40	1 (1.0)	10 (2.6)	1 (0.5)	3 (3.4)	
**Marital status**					
Married/cohabiting	88 (88.9)	324 (85.3)	180 (88.7)	75 (85.2)	0.588
Other	11 (11.1)	56 (14.7)	23 (11.3)	13 (14.8)	
**Highest education level**					
Less than high school	9 (9.1)	44 (11.6)	26 (12.8)	16 (18.2)	0.339
High school	39 (39.4)	178 (46.8)	93 (45.8)	37 (42.0)	
More than high school	38 (38.4)	112 (29.5)	53 (26.1)	27 (30.7)	
Other	13 (13.1)	46 (12.1)	31 (15.3)	8 (9.1)	
**Health literacy**^**¥¥**^					
Low	9 (9.3)	33 (8.8)	24 (12.1)	8 (9.4)	0.655
Medium	41 (42.3)	184 (49.3)	88 (44.2)	36 (42.4)	
High	47 (48.5)	156 (41.8)	87 (43.7)	41 (48.2)	
**Working status**					
Employed (not as HCP)	64 (64.6)	210 (55.4)	100 (49.3)	44 (50.6)	0.109
HCP	11 (11.1)	40 (10.6)	16 (7.9)	5 (5.7)	
Housewife	7 (7.1)	40 (10.6)	25 (12.3)	10 (11.5)	
Other	17 (17.2)	89 (23.5)	62 (30.5)	28 (32.2)	
**First language**					
Same as in country of residency	91 (91.9)	361 (95.3)	196 (96.6)	84 (96.6)	0.314
Other	8 (8.1)	18 (4.7)	7 (3.4)	3 (3.4)	
**Previous children**					
No	58 (58.6)	196 (51.6)	95 (46.8)	36 (40.9)	0.071
Yes	41 (41.4)	184 (48.4)	108 (53.2)	52 (59.1)	
Morbidity not incl. RD^**^					
Yes	21 (21.2)	71 (18.7)	46 (22.7)	19 (21.6)	0.695
No	78 (78.8)	309 (81.3)	157 (77.3)	69 (78.4)	
**Respiratory disease**^††^					
Yes	9 (9.1)	33 (8.7)	18 (8.9)	11 (12.5)	0.731
No	90 (90.9)	347 (91.3)	185 (91.1)	77 (87.5)	
**Planned pregnancy**					
Yes/ not completely unexpected	78 (78.8)	317 (83.4)	172 (84.7)	80 (90.9)	0.151
No, it was not planned	21 (21.2)	63 (16.6)	31 (15.3)	8 (9.1)	
**Folic acid use**^§§^					
Yes	84 (86.6)	322 (86.1)	167 (83.1)	68 (81.9)	0.632
No	13 (13.4)	52 (13.9)	34 (16.9)	15 (18.1)	
**Alcohol consumption during pregnancy**					
Yes	35 (35.4)	67 (17.7)	34 (16.9)	18 (20.9)	0.001
No	64 (64.6)	312 (82.3)	167 (83.1)	68 (79.1)	

HCP = health care provider; RD = respiratory disease.

^¥^One women (0.1%) did not provide information on extent of smoking in the sub-population of women smoking during pregnancy (n = 771).

Numbers may not add up to total due to missing values. For *Folic acid use* and *Alcohol consumption during pregnancy*, the response ‘cannot remember’ was treated as a missing value. Missing values are less than 5% of the total.

^*^Includes Austria, France, Italy, Switzerland, the Netherlands, United Kingdom.

^†^Includes Finland, Iceland, Norway, Sweden.

^§^Includes Croatia, Poland, Russia, Serbia, Slovenia.

^**¥¥**^Measured by a self-assessment scale comprising three questions corresponding to the set of brief screening questions (SBSQ) developed to detect inadequate or marginal health literacy in clinical settings [26,27].

^**^Includes the presence of a chronic disease (hypothyroidism, rheumatic diseases (incl. rheumatoid arthritis, psoriatic arthritis), diabetes (type I or II), epilepsy, depression, anxiety, and cardiovascular diseases (incl. hypertension, high cholesterol, and heart diseases) or others (open-ended option)).

^††^Includes the presence of asthma, allergy, rhinitis, tonsillitis, sinusitis, or other respiratory tract conditions.

^§§^Indicates folic acid use before and/or during pregnancy.

### Determinants of smoking before pregnancy

In the bivariate analysis (Table 3), women who lived in Eastern Europe, were 20 years of age or younger, lived alone, had high school or less as highest education level, had low health literacy, did not work or were housewives, had unplanned pregnancy, did not use folic acid, and consumed alcohol during pregnancy were more likely to smoke before pregnancy. Women who were older than 30 years of age, had high health literacy, were employed as healthcare personnel, and had previous children were less likely to smoke before pregnancy.

**Table 3:** Factors associated with smoking before pregnancy (n = 8344)

	**Unadjusted**		**Adjusted**	
**Maternal characteristics**	**OR**	**95% CI**	**OR**	**95% CI**
**Region of residency**				
Western Europe^*^	0.92	0.82-1.02	0.93	0.83-1.05
Northern Europe^†^	1		1	
Eastern Europe^§^	**1.91**	**1.70-2.14**	**2.25**	**1.99-2.55**
**Maternal age**				
≤ 20	**2.51**	**1.93-3.26**	1.31	0.97-1.76
21-30	1		1	
31-40	**0.65**	**0.59-0.71**	**0.82**	**0.74-0.91**
> 40	**0.49**	**0.34-0.71**	**0.57**	**0.39-0.83**
**Marital status**				
Married/cohabiting	1		1	
Other	**2.89**	**2.36-3.53**	**2.22**	**1.78-2.77**
**Highest education level**				
Less than high school	**3.57**	**2.87-4.44**	**4.23**	**3.32-5.38**
High school	**1.78**	**1.61-1.97**	**1.95**	**1.74-2.18**
More than high school	1		1	
Other	**1.30**	**1.12-1.50**	**1.48**	**1.27-1.73**
Health literacy^**^				
Low	**1.25**	**1.04-1.52**	-	-
Medium	1		-	-
High	**0.68**	**0.62-0.75**	-	-
**Working status**				
Employed (not as HCP)	1		1	
Healthcare personnel (HCP)	**0.84**	**0.73-0.97**	0.99	0.85-1.14
Housewife	0.86	0.73-1.03	**0.73**	**0.60-0.88**
Other	**1.31**	**1.16-1.47**	0.97	0.85-1.11
**First language**				
Same as in country of residency	1		-	-
Other	**0.81**	**0.06-0.99**	-	-
**Previous children**				
No	1		1	
Yes	**0.78**	**0.71-0.86**	**0.80**	**0.73-0.89**
**Morbidity not incl. RD**^††^				
Yes	1.07	0.95-1.20	-	-
No	1		-	-
**Respiratory disease**^§§^				
Yes	0.92	0.80-1.07	-	-
No	1		-	-
**Planned pregnancy**				
Yes/not completely unexpected	1		1	
No, it was not planned	**1.70**	**1.45-1.98**	**1.32**	**1.11-1.56**
Folic acid use^*†^				
Yes	1		1	
No	**1.75**	**1.49-2.05**	**1.66**	**1.40-1.97**
**Alcohol consumption during pregnancy**				
Yes	**1.27**	**1.12-1.43**	**1.36**	**1.19-1.55**
No	1		1	

The outcome variable is categorized as Smoking before pregnancy (yes = 1) and Never smoked (no = 0).

Significant p-values are in bold.

HCP = health care provider; RD = respiratory disease.

Numbers may not add up to total due to missing values. For *Folic acid use* and *Alcohol consumption during pregnancy*, the response ‘cannot remember’ was treated as a missing value. Missing values are less than 5% of the total.

^*^Includes Austria, France, Italy, Switzerland, the Netherlands, United Kingdom.

^†^Includes Finland, Iceland, Norway, Sweden.

^§^Includes Croatia, Poland, Russia, Serbia, Slovenia.

^**^Measured by a self-assessment scale comprising three questions corresponding to the set of brief screening questions (SBSQ) developed to detect inadequate or marginal health literacy in clinical settings [26,27].

^††^Includes the presence of a chronic disease (hypothyroidism, rheumatic diseases (incl. rheumatoid arthritis, psoriatic arthritis), diabetes (type I or II), epilepsy, depression, anxiety, and cardiovascular diseases (incl. hypertension, high cholesterol, and heart diseases) or others (open-ended option)).

^§§^Includes the presence of asthma, allergy, rhinitis, tonsillitis, sinusitis, or other respiratory tract conditions.

^*†^Indicates folic acid use before and/or during pregnancy.

In the multivariate analysis (Table 3), women who lived in Eastern Europe, lived alone, had high school or less as highest education level, had unplanned pregnancy, did not use folic acid, and consumed alcohol during pregnancy were more likely to smoke before pregnancy. Women who were older than 30 years of age, were housewives, and had previous children were less likely to smoke before pregnancy. Additionally, there was an interaction effect between the highest education level variable and the working status variable. Having high school as highest education level and working as healthcare personnel was positively associated with smoking before pregnancy (OR 1.63, 95% CI 1.13-2.35).

### Determinants of continuing smoking during pregnancy

Women who lived in Western Europe, were 20 years of age or younger, lived alone, had high school or less as highest education level, had low health literacy, did not work or were housewives, had previous children, had unplanned pregnancy, and did not use folic acid were more likely to continue smoking during pregnancy in the bivariate analysis (Table 4).

**Table 4:** Factors associated with continuing smoking during pregnancy (n = 2944)

	**Unadjusted**		**Adjusted**	
**Maternal characteristics**	**OR**	**95% CI**	**OR**	**95% CI**
**Region of residency**				
Western Europe^*^	**1.37**	**1.11-1.68**	**1.46**	**1.16-1.84**
Northern Europe^†^	1		1	
Eastern Europe^§^	1.19	0.97-1.46	**1.66**	**1.31-2.10**
**Maternal age**				
≤ 20	**1.46**	**1.03-2.09**	0.93	0.63-1.39
21-30	1		1	
31-40	0.97	0.81-1.16	1.19	0.97-1.45
> 40	1.72	0.90-3.30	1.82	0.91-3.65
**Marital status**				
Married/cohabiting	1		1	
Other	**2.13**	**1.63-2.77**	**1.75**	**1.30-2.35**
**Highest education level**				
Less than high school	**3.75**	**2.78-5.08**	**3.64**	**2.58-5.14**
High school	**2.58**	**2.12-3.13**	**2.53**	**2.05-3.11**
More than high school	1		1	
Other	**1.97**	**1.50-2.59**	**1.99**	**1.49-2.66**
Health literacy^**^				
Low	**1.54**	**1.13-2.09**	**1.49**	**1.08-2.06**
Medium	1		1	
High	1.00	0.84-1.19	1.06	0.87-1.28
**Working status**				
Employed (not as HCP)	1		1	
Healthcare personnel (HCP)	0.85	0.64-1.12	0.92	0.68-1.23
Housewife	**2.08**	**1.54-2.81**	**1.43**	**1.04-1.97**
Other	**1.45**	**1.18-1.77**	1.21	0.97-1.52
**First language**				
Same as in country of residency	1		-	-
Other	0.96	0.65-1.41	-	-
**Previous children**				
No	1		1	
Yes	**1.31**	**1.11-1.55**	**1.24**	**1.03-1.49**
**Co-morbidity not incl. RD**^††^				
No	1		-	-
Yes	1.19	0.97-1.47	-	-
**Respiratory disease**^§§^				
No	1		-	-
Yes	0.84	0.63-1.11	-	-
**Planned pregnancy**				
Yes/not completely unexpected	1		1	
No, it was not planned	**1.77**	**1.40-2.25**	**1.31**	**1.00-1.72**
Folic acid use^*†^				
Yes	1		1	
No	**1.74**	**1.36-2.23**	**1.59**	**1.22-2.06**
**Alcohol consumption during pregnancy**				
No	1		-	-
Yes	1.21	0.98-1.49	-	-

The outcome variable is categorized as Continuing smoking during pregnancy (yes = 1) and Quitting smoking during pregnancy (no = 0).

Significant p-values are in bold.

HCP = health care provider; RD = respiratory disease.

Numbers may not add up to total due to missing values. For *Folic acid use* and *Alcohol consumption during pregnancy*, the response ‘cannot remember’ was treated as a missing value. Missing values are less than 5% of the total.

^*^Includes Austria, France, Italy, Switzerland, the Netherlands, United Kingdom.

^†^Includes Finland, Iceland, Norway, Sweden.

^§^Includes Croatia, Poland, Russia, Serbia, Slovenia.

^**^Measured by a self-assessment scale comprising three questions corresponding to the set of brief screening questions (SBSQ) developed to detect inadequate or marginal health literacy in clinical settings [26,27].

^††^Includes the presence of a chronic disease (hypothyroidism, rheumatic diseases (incl. rheumatoid arthritis, psoriatic arthritis), diabetes (type I or II), epilepsy, depression, anxiety, and cardiovascular diseases (incl. hypertension, high cholesterol, and heart diseases) or others (open-ended option)).

^§§^Includes the presence of asthma, allergy, rhinitis, tonsillitis, sinusitis, or other respiratory tract conditions.

^*†^Indicates folic acid use before and/or during pregnancy.

In the multivariate analysis (Table 4), women who lived in Western and Eastern Europe, lived alone, had high school or less as highest education level, had low health literacy, were housewives, had previous children, had unplanned pregnancy, and did not use folic acid were more likely to continue smoking during pregnancy.

In Northern Europe the prevalence of women continuing smoking during pregnancy with less than high school as highest education level was 4.8 times higher (48.2% vs. 12.0%) than the prevalence of women with more than high school as highest education level. For Eastern and Western Europe, the differences were 2.9 times (52.0% vs. 17.8%) and 1.8 times (34.1% vs. 19.5%), respectively. In Northern Europe the prevalence of women continuing smoking during pregnancy who lived alone and had less than high school as highest education level was 4.8 times higher (54.2% vs. 11.4%) than the prevalence of women who were married or cohabiting and had more than high school as highest education level. For Eastern and Western Europe, the differences were 3.5 times (60.0% vs. 17.3%) and 1.1 times (20.0% vs. 19.0%), respectively.

### Determinants of the extent of smoking during pregnancy

There was no significant association between any maternal characteristic and the extent of smoking during pregnancy in the bivariate analysis (data not shown). In the multivariate analysis, women who lived in Eastern Europe (OR 2.07, 95% CI 1.12-3.83) and had less than high school as highest education level (OR 2.76, 95% CI 1.32-5.78) were more likely to smoke more than 10 cigarettes per day. Women who were 20 years of age or younger (OR 0.12, 95% CI 0.02-0.90) were less likely to smoke more than 10 cigarettes per day.

## Discussion

To our knowledge, this study is the first to contribute multinational comparable descriptive data on the prevalence of smoking before and during pregnancy and the extent of smoking during pregnancy in several European countries in relation to a wide range of maternal characteristics. The study is also novel in providing insights into potential determinants for smoking before pregnancy, continuing smoking during pregnancy, and the extent of smoking during pregnancy.

Several findings are important for clinical practice. First, our results are an important reminder that smoking before and during pregnancy is still prevalent in women across Europe. Currently, thousands of infants in Europe are exposed to harmful cigarette smoking during fetal life each year and therefore need continued attention from the European and national authorities. Second, detection of maternal characteristics that act as barriers to or facilitators of quitting smoking during pregnancy may assist healthcare personnel in identifying women who most likely will need tailored support and inform the important ongoing work on developing such targeted smoking cessation interventions among these women. Continuing to focus on increasing the education level among women in Europe is an important societal effort that may also result in healthier pregnancies for mother and child.

We found the highest rates of smoking before pregnancy in Eastern European countries, ranging from 46.3% in Russia to 50.0% in Croatia, as well as a more than two-fold higher risk of smoking before pregnancy in Eastern Europe compared to Northern Europe. This may be explained by the ominous shift in tobacco use in general, and in young women specifically, from high-income countries to low-and middle-income countries, driven by marketing by tobacco companies specifically targeting women [29]. Consequently, previous studies [30,31] show that this also is the case for smoking during pregnancy, with prevalence ranging from 15% in Romania to 30% in Poland. Whether or not a smoker stops smoking after learning of her pregnancy also depends on the extent of her smoking habit [32], and we found that pregnant women living in Eastern Europe were two times more likely to smoke more than 10 cigarettes per day. The prevalence of smoking during pregnancy ranged from 4.2% to 18.9% in the 15 European countries in our study. Here there was no strong trend in smoking during pregnancy status by European region; there was an approximately 1.5 times higher risk in Western and Eastern Europe compared to Northern Europe. Prevalence rates below 10% for smoking during pregnancy in most of the countries in the Northern and Western European regions (Iceland, Norway, Sweden, Austria, Italy, Switzerland, the United Kingdom) in our study support the recent decline in high-income countries from 20%-35% in the 1980s to 10%-20% in the early 2000s [12,22,33-37]. However, given the serious clinical consequences of the adverse outcomes of smoking during pregnancy, the public health impact of efficient interventions to support women to stop smoking during pregnancy is tremendous across all countries. A recent Cochrane-review [38] shows that the number of psychosocial interventions needed to treat for benefit is extraordinarily low, with approximately 71 interventions to prevent one preterm birth and 61 interventions to prevent one infant being born with low birth weight. Hence, there is a strong call for a European action plan aiming not only to reduce the prevalence of smoking during pregnancy but also to provide efficient support to the women who struggle to quit.

Our study supports previous research finding that tobacco smoking in general is strongly associated with low socioeconomic status (SES) [39], consequently increasing the health inequalities between people with high and low education and high and low incomes. We found that having less than high school as highest education level was associated with a four-fold higher risk of smoking before pregnancy. Furthermore, the more risky behaviors among smokers before pregnancy in our study – having unplanned pregnancies, no use of folic acid and alcohol use during pregnancy – was found to be related to low SES [40]. In contrast, we speculate that multiparity, being a housewife, and age older than 30 might be life-experience barriers to smoking, since the women might have quit smoking when they were expecting their last child, and they might have outgrown their smoking identity as younger independent women as they now are stay at home caregivers. The association between smoking and living alone also supports this reasoning.

We found a similar association between level of education and continuing smoking during pregnancy. There was three and a half fold increased risk of continuing smoking during pregnancy among women having less than high school as highest education level. This is in line with previous research showing that there are marked socioeconomic differences between women who continue smoking during pregnancy and those who do not [32,41]. Further, pregnant women having less than high school as highest education level were 2.76 times more likely to smoke more than 10 cigarettes per day. This supports previous research showing that the higher degree of addiction, the more difficult it is for women to quit smoking during pregnancy [42]. We also found that women 20 years of age or younger were less likely to smoke more than 10 cigarettes per day, which may indicate that the youngest women have not yet reached higher degrees of addiction. Interestingly, the continuers were more often housewives and more often lived alone, which seems to be a contradiction. However, the current literature shows that being a housewife might include the presence of unsupportive partners, economic vulnerability, and finding the caregiver-role stressful (having many previous children); hence, smoking is seen as a habituated response to these life circumstances [43]. On the other hand, the existence of a supportive non-smoking husband or another stable partner has a positive effect on the women being able to quit smoking during pregnancy. Marital status and number of children are the best investigated psychosocial factors for smoking during pregnancy, and our results are consistent with previous results showing that married women and women with no previous children have the lowest smoking prevalence [32,41]. Why having previous children was positively associated with continued smoking during pregnancy might be explained by the women’s personal previous experience of giving birth to a healthy child despite smoking during pregnancy, which creates distrust in the scientific evidence relayed by health professionals [43].

Our study showed that the women who were not able to quit smoking during pregnancy have low health literacy. This is supported by the previous finding that women with low-health literacy may ignore the detrimental effect of smoking on pregnancy outcome [44]. Previous research also shows that women with low-health literacy are more likely to have unplanned pregnancies and no use of folic acid [44], displaying more risky behaviors like the continuers in our study, who also had more unplanned pregnancies and no use of folic acid. Women who smoke during pregnancy have previously been shown to be less likely to participate in positive antenatal care, for example folic acid intake, explained by a belief that folic acid intake is not important for fetal development [40]. This underlines the detrimental effect of not understanding health information in low-health literacy women, adding to the health-compromising effects of smoking and placing the babies of pregnant smokers at a further disadvantage in utero.

In our study, there were no associations between morbidity and smoking before or during pregnancy. In the current literature, this relationship is not normally discussed, but we do not know whether this is because other studies have not found an association or have not controlled for this variable. However, there is growing concern over the strong psychological associations, especially with depression and stress [45-48].

Flemming et al. (2013) calls for recognition of the importance of understanding that why a pregnant women continues to smoke during pregnancy is closely related to the contextual factors explaining why she smoked in the first place: taking a break from daily hardships, dealing with stress, and keeping up social habits [43]. As pregnancy frequently exacerbates the barriers that made it hard to quit before pregnancy, the now even more complex circumstances reinforce the women’s dependence on smoking [41,43]. Furthermore, social pressure makes the women feel embarrassed and guilty, as they are not able to fulfill the image of the “perfect mother,” which adds so much anxiety and pressure that the women instead continue to smoke as a response or at best reduce the number of cigarettes smoked per day [41,43]. Ebert and Fahy (2007) therefore suggest that midwives take a woman-centered approach and appreciate a woman’s life situation, rather than take the current baby-centered approach focusing on preventing negative outcomes related to her “bad” behavior [41]. It is quite plausible that the women who continue smoking during pregnancy in our study live their lives in this social context, as they are educationally disadvantaged and have low health literacy, cope with daily life alone, and display more risky behaviors. Current smoking cessation efforts are either non-pharmacological strategies that use cognitive-behavioral, motivational, and supportive therapies to help women to quit (psychosocial), or pharmacological interventions, or both. The current barriers to psychosocial interventions are the challenges of translating the efficacy of research interventions into routine practice and policy, the high relapse rate postpartum, and the limited information about the effectiveness of these interventions for individual women in low- to middle-income countries, since most interventions have been developed in high-income countries [38]. For pharmacological interventions there is currently insufficient evidence to draw conclusions on the efficacy or safety of adding nicotine replacement therapy to behavioral support for smoking cessation in pregnancy [49]. It is therefore not surprising that healthcare personnel in Europe that see these women daily may struggle to give them the best possible support for smoking cessation during, and preferably also after, pregnancy. In addition, pregnant women who smoke access health services for pregnancy-related issues, not smoking issues, and might therefore feel criticized when the focus suddenly is on their “bad” behavior. And labelling smoking in pregnancy solely as a social problem may make health professionals reluctant to intervene and offer support [50].

### Strengths and limitations

An important strength of this study is that data collection was performed uniformly across all participating countries. Utilization of the same questionnaire on a multinational level allows for inter-country comparison of the prevalence of smoking, continuing smoking during pregnancy, and the extent of smoking during pregnancy. Additionally, women may feel more comfortable in answering sensitive questions truthfully on an anonymous, web-based questionnaire as compared to in a face-to-face interview with a researcher/health care provider. Further, the utilization of a web-based questionnaire allowed us to reach a large proportion of the birthing population in several European countries. However, we cannot exclude the possibility that the participating women differed from the general birthing population in other ways than our analysis could control for. For example, in France, the Netherlands, and Russia the study sample was a small proportion of the general birthing population, thus the generalizability of our findings for these specific countries should be considered when interpreting the results. In addition, having information about smoking status prior to pregnancy made it possible to calculate the “true” rate of cessation, as the proportion of quitters is based on the sample of smokers before pregnancy and not the total sample of both smokers and non-smokers before pregnancy.

One limitation of the study is that the information about smoking is based on self-reported data from two constructed smoking questions. More advanced validated psychometric instruments such as the Fagerstrom Test for Nicotine Dependence [51], the Nicotine Dependence Syndrome Scale [52], and the cigarette dependence scale [53] are available for measuring dependence. In the main study that our study is based on [25], these instruments were considered too lengthy to be included. In addition, using a biochemical marker such as cotinine level in saliva, urine, or blood to confirm self-reported smoking status would have been useful, as smoking status based only on self-report show trends of underestimation [54]. Furthermore, all variables are dependent on the woman’s perception, accuracy, and recall. For new mothers, data were registered retrospectively; hence, an additional recall bias for this group of women cannot be ruled out. Stratified analyses show that in the total sample (n = 8344) reporting of alcohol consumption during pregnancy differed significantly (Pearson Chi square test p ≤ 0.001) between pregnant women and new mothers; however, the prevalence was higher for new mothers, which does not support such a bias. In the sample of smokers before pregnancy (n = 2944) reporting of respiratory disease differed significantly (Pearson Chi square test p = 0.006) between pregnant women and new mothers, but again, the prevalence was higher for new mothers, which does not support such a bias. Inclusion of pregnant women at any gestational week could have had an impact on the prevalence of women smoking during pregnancy; women at an early stage might have not stopped smoking at the moment of completing the questionnaire but might have done so later in the pregnancy. However, there were no significant differences in the reporting of smoking during pregnancy between pregnant women and new mothers or between pregnant women in their first, second, or third trimester. On the other hand, the participants may have underreported their actual smoking status irrespective of gestational week due to the sensitivity of the question. The questionnaire was only available through Internet websites; therefore, a conventional response rate could not be calculated. However, recent epidemiological studies indicate reasonable validity of web-based recruitment [55,56]. Also, in Europe the penetration rate of Internet in households or at work is relatively high among women of childbearing age, from about 50% in Russia and Serbia to 100% in Iceland [57,58]. Hence, the degree to which our findings can be extrapolated to the target population is based on the representativeness of the respondents to the general birthing population in the countries. On average, the women in the study had higher education and were slightly more often primiparous than the general birthing population in each country. In addition, the scale used to assess health literacy has not been validated in a population of reproductive young women like the population in our study. Lastly, this study did not include potentially important determinants, such as income, amount smoked before pregnancy, the presence of other household smokers, and information about nicotine replacement therapy. These advantages and limitations should be kept in mind when interpreting our results.

## Conclusions

We found the highest rates of smoking before pregnancy in Eastern European countries, and a prevalence of smoking during pregnancy ranging from 4.2% to 18.9% in the 15 European countries in the study. Women with fewer resources living in Western or Eastern Europe are more likely to continue smoking during pregnancy. They are characterized as living alone, having high school or less as highest education level, having low health literacy, being a housewife, having previous children, having unplanned pregnancy, and no use of folic acid. These findings have implications for antenatal care of women in Europe. Given the clear significance of the entirely preventable risk factor for adverse fetal outcomes that smoking during pregnancy is, knowledge about high-risk groups of women are essential for designing preventive and interventional efforts at a European and national level. Maternity health care personnel should be made aware of this high-risk group of women and manage their care accordingly. Furthermore, health policy makers and maternity health care personnel should acknowledge that although effective interventions to promote smoking cessation in pregnancy exists on research basis, the context of the women’s lives makes it difficult for them to quit and to remain non-smokers.

## Competing interests

The authors declare that they have no competing interests.

## Authors’ contributions

HN and AL conceived the study design and conducted the study. JS analyzed and interpreted the data and prepared the manuscript. HN, AL, and ACM were involved in drafting and critically revising the manuscript. All authors read and approved the final version.

## Authors’ information

JS Dr. Philos., MSc. Pharm. is a post-doctoral researcher at the School of Pharmacy, University of Oslo, Oslo, Norway. This study was a part of her post-doctoral research project. AL MSc. Pharm. is a PhD student at the School of Pharmacy, University of Oslo, Oslo, Norway. The focus of her research is the safety of medications in pregnancy, antidepressants during pregnancy, pharmacoepidemiology, and mental health during pregnancy. ACM Dr. Med Sci., MSc. Pharm. is a project manager at Sahlgrenska University Hospital, Gothenburg, Sweden. The focus of her research is health-related behaviors in different populations from a gender perspective and with epidemiological methods. HN Dr. Philos., MSc. Pharm. is a professor at the School of Pharmacy, University of Oslo, Oslo, Norway and a researcher at the Division of Mental Health, Norwegian Institute of Public Health, Oslo, Norway. The focus of her research is medication use and safety during pregnancy and breastfeeding.

## Pre-publication history

The pre-publication history for this paper can be accessed here:

http://www.biomedcentral.com/1471-2393/14/213/prepub
